# Wildfires and climate justice: future wildfire events predicted to disproportionally impact socioeconomically vulnerable communities in North Carolina

**DOI:** 10.3389/fpubh.2024.1339700

**Published:** 2024-04-29

**Authors:** Raquel Winker, Alexis Payton, Eric Brown, Elena McDermott, Jonathan H. Freedman, Chris Lenhardt, Lauren A. Eaves, Rebecca C. Fry, Julia E. Rager

**Affiliations:** ^1^Department of Environmental Sciences and Engineering, University of North Carolina, Chapel Hill, NC, United States; ^2^Institute for Environmental Health Solutions, Gillings School of Global Public Health, University of North Carolina, Chapel Hill, NC, United States; ^3^Center for Environmental Medicine, Asthma and Lung Biology, School of Medicine, University of North Carolina, Chapel Hill, NC, United States; ^4^Renaissance Computing Institute (RENCI), University of North Carolina, Chapel Hill, NC, United States; ^5^Curriculum in Toxicology and Environmental Medicine, School of Medicine, University of North Carolina, Chapel Hill, NC, United States

**Keywords:** wildfire, environmental justice, social vulnerability, housing cost, cluster analysis, climate change

## Abstract

Wildfire events are becoming increasingly common across many areas of the United States, including North Carolina (NC). Wildfires can cause immediate damage to properties, and wildfire smoke conditions can harm the overall health of exposed communities. It is critical to identify communities at increased risk of wildfire events, particularly in areas with that have sociodemographic disparities and low socioeconomic status (SES) that may exacerbate incurred impacts of wildfire events. This study set out to: (1) characterize the distribution of wildfire risk across NC; (2) implement integrative cluster analyses to identify regions that contain communities with increased vulnerability to the impacts of wildfire events due to sociodemographic characteristics; (3) provide summary-level statistics of populations with highest wildfire risk, highlighting SES and housing cost factors; and (4) disseminate wildfire risk information via our online web application, ENVIROSCAN. Wildfire hazard potential (WHP) indices were organized at the census tract-level, and distributions were analyzed for spatial autocorrelation via global and local Moran’s tests. Sociodemographic characteristics were analyzed via *k*-means analysis to identify clusters with distinct SES patterns to characterize regions of similar sociodemographic/socioeconomic disparities. These SES groupings were overlayed with housing and wildfire risk profiles to establish patterns of risk across NC. Resulting geospatial analyses identified areas largely in Southeastern NC with high risk of wildfires that were significantly correlated with neighboring regions with high WHP, highlighting adjacent regions of high risk for future wildfire events. Cluster-based analysis of SES factors resulted in three groups of regions categorized through distinct SES profiling; two of these clusters (Clusters 2 and 3) contained indicators of high SES vulnerability. Cluster 2 contained a higher percentage of younger (<5 years), non-white, Hispanic and/or Latino residents; while Cluster 3 had the highest mean WHP and was characterized by a higher percentage of non-white residents, poverty, and less than a high school education. Counties of particular SES and WHP-combined vulnerability include those with majority non-white residents, tribal communities, and below poverty level households largely located in Southeastern NC. WHP values per census tract were dispersed to the public via the ENVIROSCAN application, alongside other environmentally-relevant data.

## Introduction

Wildfires are an increasing threat to public health across the world. Globally, the frequency and intensity of wildfires continues to increase in association with climate change factors (1). Wildfires have occurred throughout the United States (US), particularly along the West coast and certain regions of the Southeast including North Carolina (NC) (2). Historically, large wildfires have not been as common in NC in comparison to states such as California and Oregon; though as climate change exacerbates drought conditions, wildfires in NC are predicted to become a greater threat (3). In the future, wildfires in NC are expected to inflict greater damage to human health and infrastructure, as both the fire season and the average area burned are expected to increase as a result of global climate change (4). NC’s landscape is especially conducive to wildfire events due to the large area covered by burnable forests and the growing development of the wildland-urban interface (WUI) (4). Between 1985 and 2016, large amounts of land were burned in NC wildfires during periods that coincided with droughts in the state, a trend that will only increase as severe droughts likely become more frequent and intense as climate change progresses (4, 5). NC’s wildfires demonstrate a unique risk to human health, because they vary greatly in smoke composition, intensity, and burn time. These variable conditions makes wildfire exposures and resulting impacts on health less predictable (6). Additionally, wildfires can impact extensive areas of land due to traveling smoke plumes dictated by wind and weather patterns (7). Wildfire smoke contains a diverse range of air toxicants that can impact human health, such as particulate matter, hydrocarbons, ozone, sulfur dioxide, nitrogen dioxide, inorganics, and ionic constituents (8). Exposure to wildfire smoke has thus been associated with a variety of adverse health outcomes, including increased risk of asthma exacerbation, chronic obstructive pulmonary disease (COPD), respiratory infections, and all-cause mortality (9).

In NC, as across the US, historical and current practices and policies of racialized economic isolation and exclusion result in low-income and Black and Indigenous People of Color (BIPOC) communities being disproportionately faced with environmental threats (10, 11). Environmental justice recognizes both these disproportionate exposures and disparities in access to resources to recover and build resiliency in the face of such hazards (12). Climate change and its effects, including flooding, high temperatures and wildfire, represent an evolving environmental hazard with disproportionate impacts (13, 14). In fact, in NC, wildfire related health outcomes including asthma and COPD have well-documented racial disparities in prevalence (15, 16). Recognition and analysis of these trends within the frameworks of both science and environmental justice has led to a more recently developed framework known as climate justice, which highlights the connection between climate change and social inequalities (17, 18). Data-driven analyses are needed to identify communities at greatest risk of experiencing negative economic and health impacts due to climate change hazards, such as wildfire events.

Throughout the US, communities of low socioeconomic (SES) have been identified as having higher risk of experiencing wildfire events and suffering the subsequent health and economic effects, such as impacts on housing, highlighting that wildfires are a climate justice issue (19–21). There are two routes by which environmental hazards impact communities and lead to inequities: differential exposure and differential susceptibility (also referred to as vulnerability) (22). Socially vulnerable populations are not necessarily exposed to wildfire smoke more than populations that are less socially vulnerable; instead, wildfire events can induce greater effects on the health and well-being of populations with increased social vulnerability (21). This is in large part due to a lack of resources within marginalized and/or low-income communities that are crucial to the adaptation and recovery from wildfire events. Resources needed include education on wildfire risk, proper insurance, funds to rebuild and repair damages, and access to healthcare after smoke inhalation-induced health consequences (20). Indeed, previous research has found that when comparing cardiopulmonary risk due to wildfire exposure, the largest difference in low-risk vs. high-risk communities is SES factors (23).

This project aimed to identify communities that are most likely to experience the negative effects of wildfire events by examining regions that exhibit both socioeconomic vulnerability and high wildfire risk throughout the state of NC. We modeled this analysis after the Chemical and Social Stressors Integration Technique (CASS-IT), a methodology to identify regions of holistic public health concern based on environmental and social exposures (24). Further, this project sought to disseminate wildfire risk information alongside social stressor data through our expanding web application, ENVIROSCAN (25). NC was selected as a population of interest due to its vast heterogeneity in terms of SES factors, geography and vegetation, its history of wildfire events and because it has the largest number of wildland-urban interface acres (areas of greatest vulnerability to wildfire effects) in the US (26–28). Leveraging the unique metric of Wildfire Hazard Potential (WHP) and integrative clustering analyses, this study provides a unique perspective on the potential impacts of future wildfire events and highlights climate justice concerns in NC.

## Methods

### Wildfire risk values

Different indices can be utilized to assess a geographical area’s risk of future wildfires; one of the most recognized is Wildfire Hazard Potential (WHP) (29). WHP is an index compiled by the United States Department of Agriculture (USDA) that quantifies the relative potential for a future wildfire event, based upon the combination of multiple variables that contribute to the overall risk and hazard of wildfires occurring. In the contiguous US, these values range from 0 to 95,415 (29). This index specifically takes into account landscape conditions (e.g., vegetation and wildland fuel types) that inform burn probability modeling simulations for large wildfire events, as well as historic small wildfire occurrence data. The specific WHP values used for this study were obtained from the most recent version of the USDA’s Forest Service’s WHP Index updated in 2020. The underlying methods surrounding the derivation of WHP values have been previously reported (29).

To organize the WHP data for the current analysis, values originally provided at the county-level for all 100 NC counties were converted to census-tract level for all 2,195 census tracts. This conversion was needed to integrate WHP values with various SES and housing variables in downstream analyses and also to provide greater resolution and regional variation within the data. In completing this conversion, census tract data were sourced from the American Community Survey (30). WHP values were then calculated for each census tract by calculating the WHP average of the county or counties that overlapped within that tract, weighted according to the covered area, in ArcMap v. 10.8.2.

### SES and housing data

Socioeconomic and sociodemographic data at the census tract level were obtained from the most current American Community Survey 5-year estimates (from 2010) (30). From these data, environmental justice screening (EJScreen) variables, as previously identified by the United States Environmental Protection Agency (US EPA), were derived as general markers to assess potential vulnerability to environmental pollution (31). These variables included the following socioeconomic data: less than high school education, unemployment rate, and population in poverty. Variables also include the following sociodemographic data: population over 65, population under 5, people of color, and non-proficient English-speaking population. Ethnicity (i.e., Hispanic or non-Hispanic) was incorporated as an additional variable alongside the EJScreen indicators. We refer to these eight variables as “SES variables” throughout this analysis. As a further means to evaluate impacts of future wildfire events on vulnerable populations, housing data were organized at the census-tract level and were accessed via the Social Explorer website drawing from the US Census (32). Housing variables included housing density and median house value. All SES and housing-level data were available across the entire U.S. and filtered here for census tracts within NC.

### Data processing and missing value imputation

All data processing and subsequent analyses were carried out in R software (v4.1.2.). Background filters were applied to the WHP, SES, and housing data, separately. These filters included the removal of variables and/or census tracts in NC with <25% of data, resulting in the following: for the WHP values, wildfire hazard data were retained across all 2,195 census tracts. For the SES data, records of missing data resulted in the removal of 25 census tracts that contained information on <25% of the eight SES variables yielding inclusion of 2,170 census tracts. For the housing data, records of missing data resulted in the removal of 22 census tracts, yielding the inclusion of 2,173 census tracts. Lastly, data imputation was performed to generate values for the remaining missing data through the *missForest* package which uses random forest modeling to predict data points for missing values (33). Missing values were imputed on a per-variable distribution basis, yielding complete information for the 2,195 tracts for WHP; 2,169 tracts for SES; and 2,173 tracts for housing, making the integrative analyses described below focus on the 2,169 tracts with complete information.

### WHP distribution analysis across NC

NC shapefiles, simplified formats for storing geographic information, were downloaded in R using the *tigris* package (34) at the county and census tract levels, and data were merged with the WHP data. These two levels of granularity allowed for visualizations of WHP by county and census tract, which were also summarized by overall average and by quintile to provide a more comprehensive landscape of WHP across NC. Geographic visualizations of these summary-level WHP distributions were produced using the *ggplot2* package (35).

Spatial autocorrelation tests were carried out to assess how correlated each WHP value was in relation to nearby geographic locations. Here, global and local Moran’s tests were performed using the *spdep* package (36). The global Moran’s test assessed correlation of WHP across the entire state. The local Moran’s test assessed correlation between each individual census tract and census tracts that share a border or vertex, known as contiguity-based neighbors (37). Resulting *p*-values from the local Moran’s tests were adjusted for multiple tests (referred to as “P adjusted” or “p_adj_” values) based on false discovery rate (FDR) q-values (38). Correlations from the local Moran’s tests were categorized into quadrants: (1) low to low, defined as areas with low WHP significantly correlated with contiguity-based neighbors with low WHP; (2) low to high, defined as areas with low WHP significantly correlated with contiguity-based neighbors with high WHP; (3) high to low, defined as areas with high WHP significantly correlated with contiguity-based neighbors with low WHP; and (4) high to high, defined as areas with high WHP significantly correlated with contiguity-based neighbors with high WHP. Throughout these quadrant definitions, the local census tract was defined to have low WHP if its WHP value was less than the average WHP across all of NC (WHP < 139.24) and high WHP if its value was greater than the average WHP across all of NC (WHP > 139.24). The contiguity-based neighbors were defined to have low WHP if the local Moran’s statistic was less than global Moran’s statistic (0.92) and high WHP if the local Moran’s statistic was greater than the global Moran’s statistic. Significant correlation was defined as p_adj_ < 0.05. The remaining correlation pairs were categorized as insignificant. These results were mapped onto North Carolina highlighting regions that were significantly autocorrelated. To provide further clarification, all categories for spatial autocorrelation results are summarized in Table 1.

**Table 1:** Categorized results of spatial autocorrelation analysis of WHP values across NC, per census tract.

Local autocorrelation category	WHP category in primary census tract of focus^1,2^	WHP category in continuity-based neighbor census tracts^1,2^	Autocorrelation significance	Number of NC census tracts derived per category
High to high	High	High	p_adj_ < 0.05	311
High to low	High	Low	p_adj_ < 0.05	7
Low to high	Low	High	p_adj_ < 0.05	9
Low to low	Low	Low	p_adj_ < 0.05	10
Insignificant	-	-	p_adj_ ≥ 0.05	1858

Spatial autocorrelation was analyzed using a local Moran’s test. ^1^Primary census tract of focus was defined as having low WHP if its WHP value was less than the average WHP across all of NC per census tract (<139.24) and high WHP if its WHP was greater than or equal to the average WHP for NC. ^2^Contiguity-based neighbor census tracts were defined as having low WHP if the local Moran’s statistic was less than the global Moran’s statistic (<0.92) and high if the local Moran’s statistic was greater than or equal to the global Moran’s statistic.

### Integrative cluster analysis across SES factors to identify socially vulnerable communities at risk of wildfires

An integrative cluster analysis was carried out to determine whether distinct groups (“clusters”) of census tracts could be derived using SES variables relevant to the EJScreen (31). The goal was to visualize these distinct SES profiles and overlay them with housing and WHP factors to quantify the impacts and risks of wildfires within socially vulnerable communities. This method notably parallels our recent data integration and visualization technique for analyzing chemical and social stressor information (24); though here, instead of chemical stressors we utilize wildfire risk information.

To identify distinct regions throughout NC based upon SES profiles, *k*-means clustering was employed. Prior to analysis, data were standardized using the *scale* function in base R. *K*-means, an unsupervised machine learning technique (39, 40), was used to find patterns between census tracts based on SES data distributions. Cluster assignments were derived by minimizing within cluster differences and maximizing between cluster differences using the *factoextra* package in R (41). *K* refers to the number of clusters and the centroid represents the average of the points assigned to the cluster. The optimal cluster number was selected using two approaches: the first being the elbow method, where the optimal cluster number is where the within cluster variation is minimized. The second approach consisted of comparing two-dimensional principal component plots to assess the cluster number that best separated the census tracts (42, 43). Resulting clusters were visualized on a map of NC. To quantify the magnitude and directionality of each variable’s impact on the clusters, the average of each variable’s value (scaled for visualization and unscaled for cluster-level reporting) was calculated per cluster. Housing and WHP characteristics were then summarized on a per-cluster basis and assessed for significant differences across multiple clusters using an ANOVA test followed by Tukey’s *post hoc* tests. This approach yielded summary-level information on SES profiles, housing units, and WHP, to identify groups of socially vulnerable communities most at-risk for future wildfire events.

### Evaluation of NC communities with the highest WHP (>95th percentile)

A focused analysis was carried out on regions that were at highest risk of future wildfire events, relative to other areas of NC. In this analysis, a filter was applied, in which census tracts that fell above the 95th percentile for WHP were considered to have the highest risk of future wildfire events. The cut-off of the 95th percentile was selected to parallel previous records of wildland risk profiles across the U.S. (44). Summary-level SES and housing data statistics were calculated for these high-risk areas to characterize the population most likely to be affected by future wildfire events in NC. This information was also converted into an infographic for communication purposes via BioRender Software.

### Dissemination of WHP information to the public via ENVIROSCAN web application

ENVIROSCAN is an online application that enables web users to visualize and compare socioeconomic and environmental health indicators (25). The ENVIROSCAN project was led by researchers and web developers within the University of North Carolina at Chapel Hill Superfund Research Program (UNC-SRP), UNC Institute of Environmental Health Solutions (IEHS), and the Renaissance Computing Institute (RENCI). This web application allows users to filter by certain SES variables (e.g., race, percent poverty, and percent multilingual) and view these alongside environmental exposure/health risk information, such as metals contamination in drinking water (45). The current study organized the upload and visualization of WHP data into this publicly available web application for easy observation and information regarding wildfire risk throughout NC. Further instruction can be found within the NC ENVIROSCAN “Mapper Cheat Sheet” (46).

## Results

### Distributions of wildfire risk values across NC

**Figure 1:**
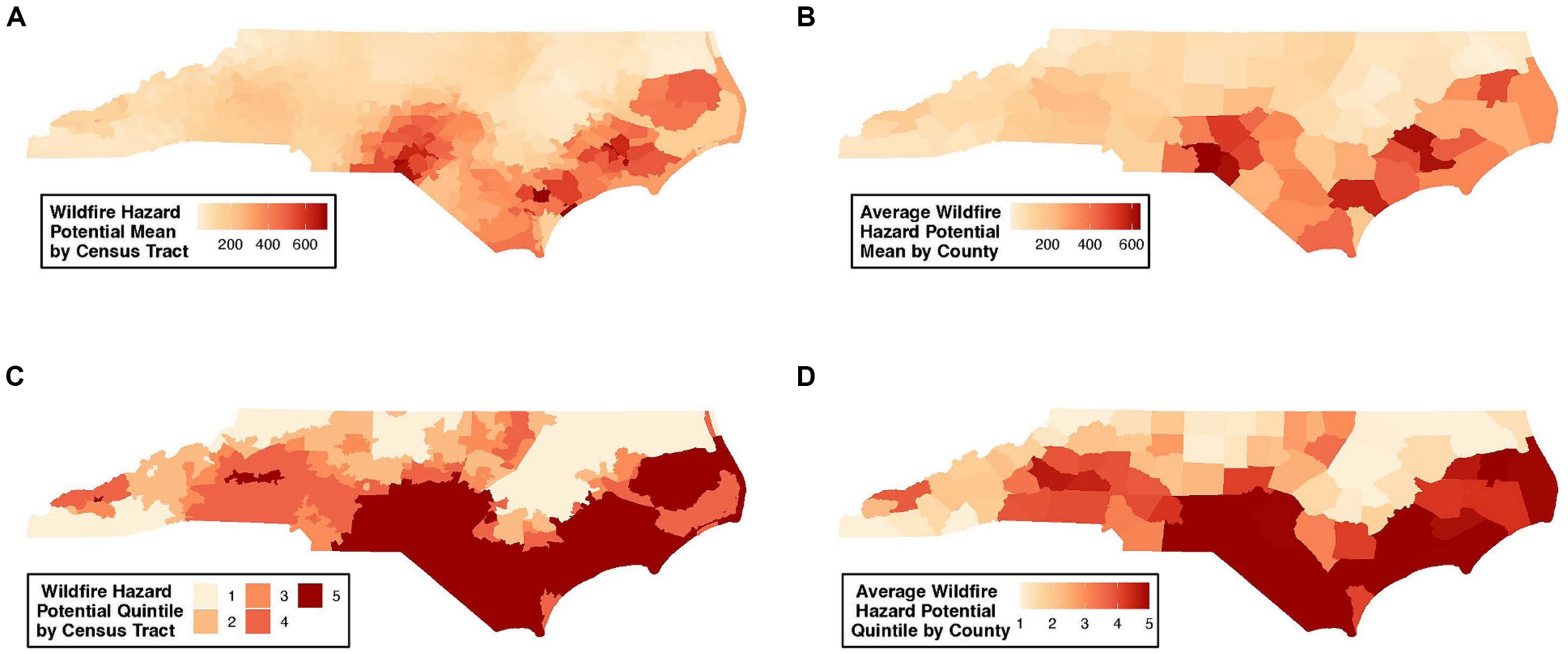
Geospatial distribution of the risk of future wildfire events across NC. Wildfire risk is presented as wildfire hazard potential (WHP) values, summarized as: **(A)** WHP values per census tract; **(B)** averaged WHP values per county; **(C)** WHP quintile per census tract; **(D)** averaged WHP quintile per county.

WHP varied considerably across NC, with the highest WHP values localized in the Southeastern region of the state (Figure 1). The overall WHP values, per census tract, had a mean of 175 and median of 132. The distribution of census tract-level WHP values per quintile spanned the following: 1st quintile = 22 (overall min)—93; 2nd quintile = 93–121; 3rd quintile = 121–139; 4th quintile = 139–222; 5th quintile = 222–716 (overall max). All WHP values averaged per census tract and county are provided in Supplementary Tables S1, S2. The three counties with the highest average WHP values included Richmond (mean WHP = 640), Scotland (610), and Craven (607) counties, highlighted in Figure 1, followed by Pender (565), Moore (522), and Hoke (495) counties. The three counties with the lowest average WHP values included Pasquotank (mean WHP = 26), Perquimans (35), and Edgecombe (41) county (Supplementary Table S2).

**Figure 2:**
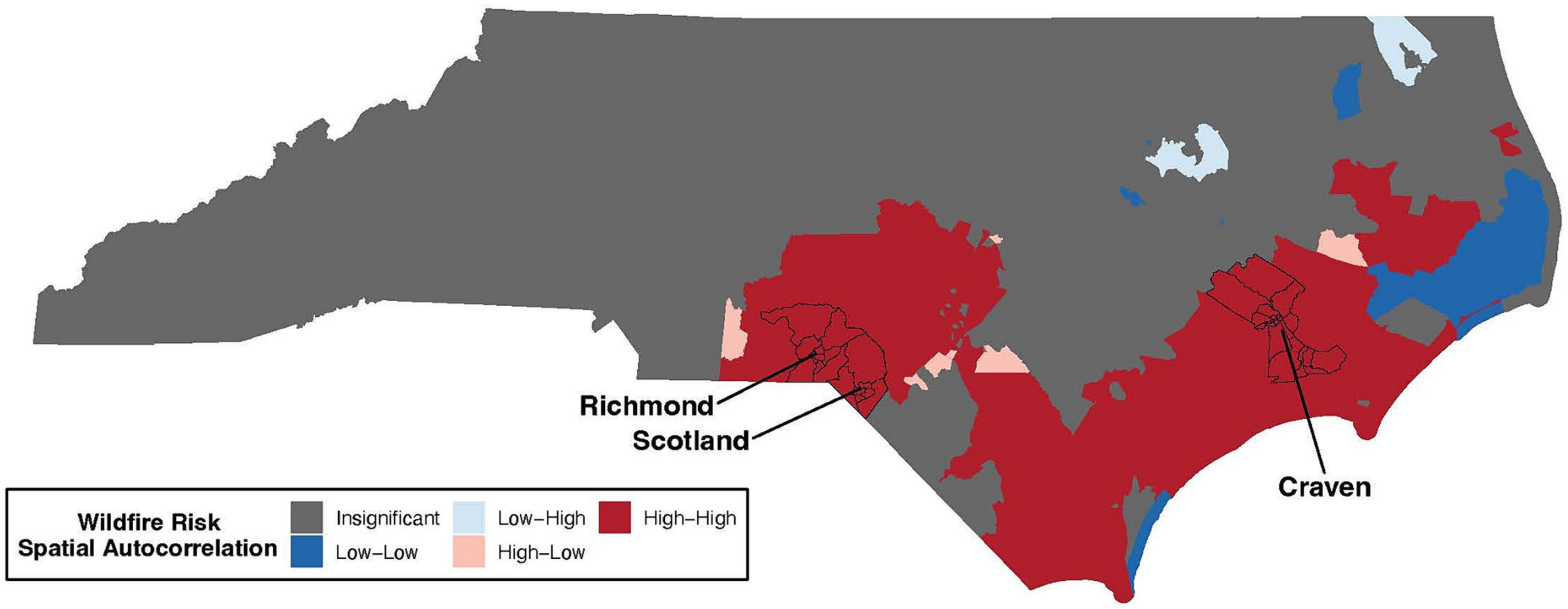
Wildfire risk spatial autocorrelation across regions of NC. Census tracts with WHP values significantly correlated with continuity-based neighbors are shaded in red or blue, while census tracts with insignificant correlations are shaded in gray. Detailed definitions of autocorrelation categories provided in the methods and reiterated in Table 1. The counties with the strongest degree of spatial autocorrelation (Richmond, Scotland, and Craven) are labeled and their corresponding census tracts are outlined in black.

Tract-level WHP values were used to identify regions of NC with high vs. low levels of spatial autocorrelation of wildfire risk. Globally, there was significant (*p* = 2.2×10^−16^) WHP spatial autocorrelation across the state (global Moran’s statistic = 0.92), while locally there was significant (p_adj_ < 0.05) high to high spatial autocorrelation in 337 census tracts primarily concentrated in Southeastern NC (Figure 2). Richmond, Scotland, and Craven counties contained census tracts with the strongest degree of spatial autocorrelation. This finding supports that a localized area of high WHP exists in the Southeastern regions of NC. Other categories of local WHP spatial autocorrelation included a small number of counties with high to low; low to high; and low to low spatial autocorrelation, with most census tracts showing insignificant spatial autocorrelation (Table 1). All spatial autocorrelation statistics are detailed in Supplementary Table S3.

### Distributions of SES and housing data across NC

Distributions of SES variables, including education, unemployment, poverty, age, people of color, and non-proficient English-speakers, varied considerably across NC. For example, percent poverty ranged from 2.8% to 36.5% (5th to 95th%) per census tract. In addition, the percentage of the population that identifies as non-White ranged from 3.5% to 78.7% (5th to 95th%). Summary-level statistics for all SES variables included in this analysis are provided in Table 2A, and all values per census tract are detailed in Supplementary Table S4. Housing variables also varied across NC, with housing density ranging from 15.3 to 2527.2 (5th to 95th%) units per square mile. Median house value ranged from $73,100.00 to $398,560.00 (5th to 95th%). Further summary statistics for housing variables are included in Table 2B.

**Table 2:** Summary statistics for **(A)** SES and **(B)** housing indicators across NC census tracts.

Variables	Mean	Standard deviation	5th percentile	95th percentile
**(A) SES variables**				
Hispanic/Latino (%)	9.10	8.49	0.70	26.16
Less than high school (%)	26.20	17.37	0.00	55.40
Non proficient English speakers (%)	4.41	5.15	0.00	14.30
Population over 65 (%)	16.68	7.58	6.30	29.50
Population under 5 (%)	5.75	2.58	2.20	10.00
Poverty Overall (%)	15.81	10.60	2.80	36.46
Race Non White (%)	31.19	23.57	3.45	78.72
Unemployed (%)	5.98	4.52	1.25	13.38
**(B) Housing indicators**				
Housing density (units/ sq. mile)	642.84	1051.90	15.25	2527.15
Median house value ($)	170,422.15	106,593.12	73,100.00	398,560.00

Statistics include mean, standard deviation, 5th percentile, and 95th percentile.

### Integrative regional clusters based upon SES factors highlights socially vulnerable communities

**Figure 3:**
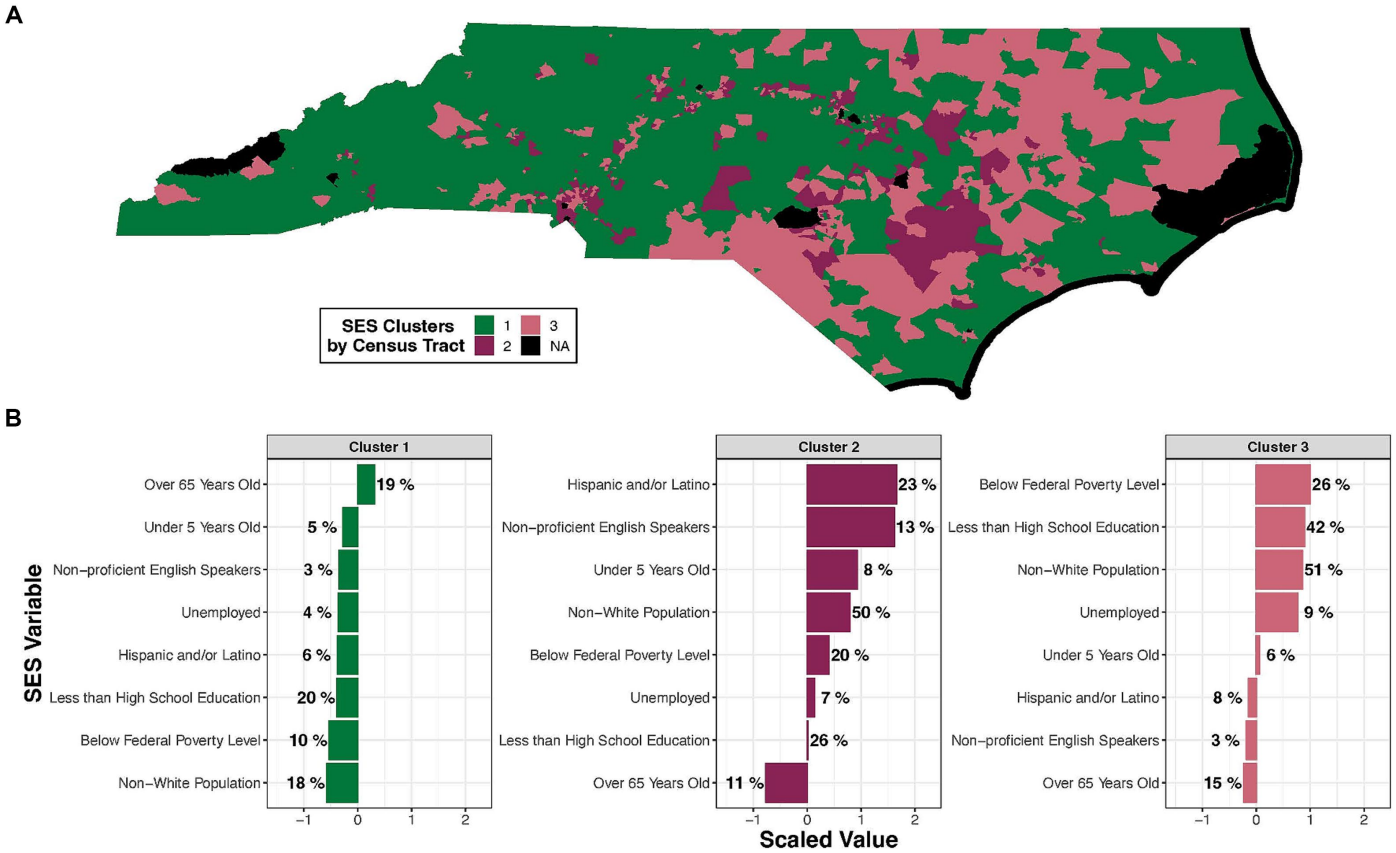
K-means cluster analysis of SES factors across NC highlights distinct clusters of vulnerability to the consequences of wildfire events. **(A)** Three SES clusters are visualized across NC. Census tracts without complete SES data are shown in black. **(B)** The value of each SES variable is displayed per cluster. Each variable’s value was scaled across the clusters, averaged within each cluster, and is displayed on the x axis. Averaged raw values are displayed as percentages next to each bar. SES variables are organized on the y axis from the highest to lowest scaled value.

In deriving clusters of census tracts with similar SES profiles, the first step was to identify the optimal number of clusters. A final *k* value of 3 (denoting the number of clusters) was selected as producing the most distinct groupings across SES factors. All census tracts are illustrated according to SES-based clusters in Figure 3A, with corresponding SES values for each cluster in Figure 3B and Supplementary Table S5. Each census tract’s cluster assignment and variable value are detailed in Supplementary Table S4.

In summary, this analysis of the SES variables indicated one cluster of NC census tracts with less SES vulnerability and two clusters with more SES vulnerability. Specifically, “Cluster 1” is characterized by an older (20% of the population over 65 years old), mainly White (18% of the population identifying as non-White), population with a lower percentage (20%) of residents with less than a high school education. “Cluster 2” is characterized by a high percentage of younger (8% < 5 years of age), non-White (50%) and Hispanic and/or Latino (23%) residents with a lower percentage (26%) of residents who have less than a high school education. “Cluster 3” is characterized by a high percentage (51%) of non-White residents and a high prevalence of poverty (26%), as well as a substantial portion of the population without a high school education (42%). Clusters 2 and 3 were thus identified as areas containing socially vulnerable populations. These two groups were more concentrated in Eastern NC (Figure 3A).

**Figure 4:**
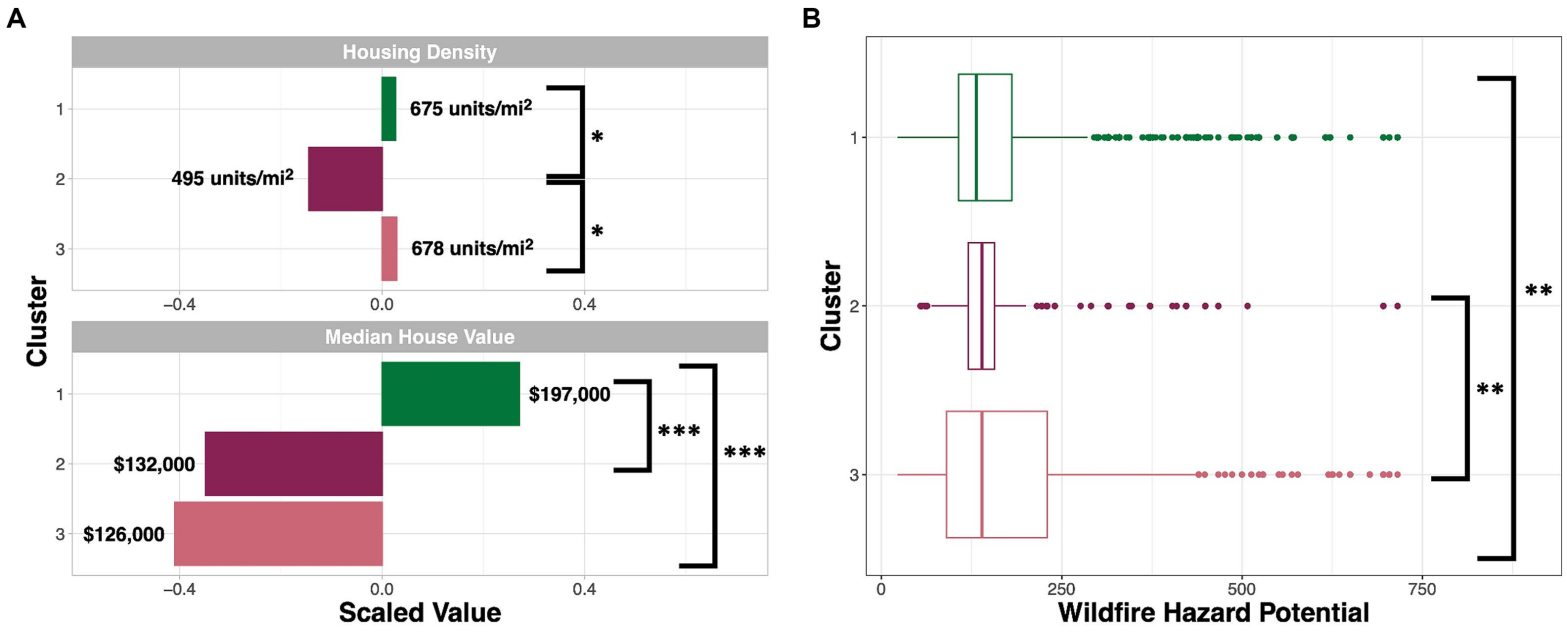
Cluster-level values of **(A)** housing variables and **(B)** WHP across NC. **(A)** The value of each housing variable is displayed per SES cluster. Each variable’s value was scaled across the clusters, averaged within each cluster, and is displayed on the x axis. Averaged raw values are displayed next to each bar. **(B)** Average WHP per cluster is shown on the x axis. Comparisons that were significant based on Tukey’s *post hoc* tests include the following for the displayed two-group comparisons: ^*^padj < 0.05, ^**^padj < 0.01, ^***^padj < 0.001. All multi-group comparisons within variable type (i.e., housing density, median house value, and WHP) also demonstrated significance (padj < 0.05).

In terms of housing indices, Cluster 1, the cluster characterized largely by an older, White, and educated population, notably has the greatest median house value of approximately $197,000 (Figure 4A). In comparison, Clusters 2 and 3 had median house values of $132,000 and $126,000, respectively and were both significantly different (p_adj_ < 0.05) from Cluster 1. Cluster 2, characterized largely by a younger, Hispanic and/or Latino population, had the lowest housing density with 495 properties/mi^2^ compared to Cluster 1 and Cluster 3 with 675 and 678 units/mi^2^, respectively. Cluster 1 was significantly different (p_adj_ < 0.05) from Cluster 2; and Cluster 3 was significantly different (p_adj_ < 0.05) from Cluster 2 in terms of housing variables (Figure 4A; Supplementary Table S6). When evaluating WHP in a multigroup comparison, all clusters showed significantly different WHP values (p_adj_ < 0.05). In pairwise comparisons, Cluster 1 (mean WHP of 170) was significantly different (p_adj_ < 0.05) from Cluster 3 (mean WHP of 189); and Cluster 3 was significantly different from Cluster 2 (mean WHP of 165; Figure 4B; Supplementary Table S6).

### Summary level statistics for NC communities with the highest WHP (>95th percentile)

A total of 102 census tracts were found to have WHP of at least 439 (95th percentile), and thus were considered to be at greatest risk for future wildfire events. To summarize the potential impacts of the future wildfire events, the following summary-level statistics were derived:

- Over 200,000 homes, worth $31billion, are located in 102 census tracts with elevated risk to future wildfire events (based upon WHP > 95th percentile).
- Approximately 1 in 3 residents in these high-risk regions were people of color and/or those that have less than a high school education, representing sociodemographic and socioeconomic vulnerability.
- 100 out of these 102 high risk census tracts were significantly correlated with neighboring regions with high WHP risk, highlighting adjacent regions of vulnerability.
- From the integrative cluster analysis, ~50% (49 of 102) of the census tracts at highest risk of future wildfire events (WHP > 95th percentile) had SES-based vulnerability, being located in either Cluster 2 or 3.
- Almost half of the 200,000 homes located in areas with elevated wildfire risk were in SES-based vulnerable census tracts but are only worth about a third of the total value of the high-risk properties ($10.4B out of the $31B total value of homes at highest wildfire risk with an average value of ~$100,000 per home).

**Figure 5:**
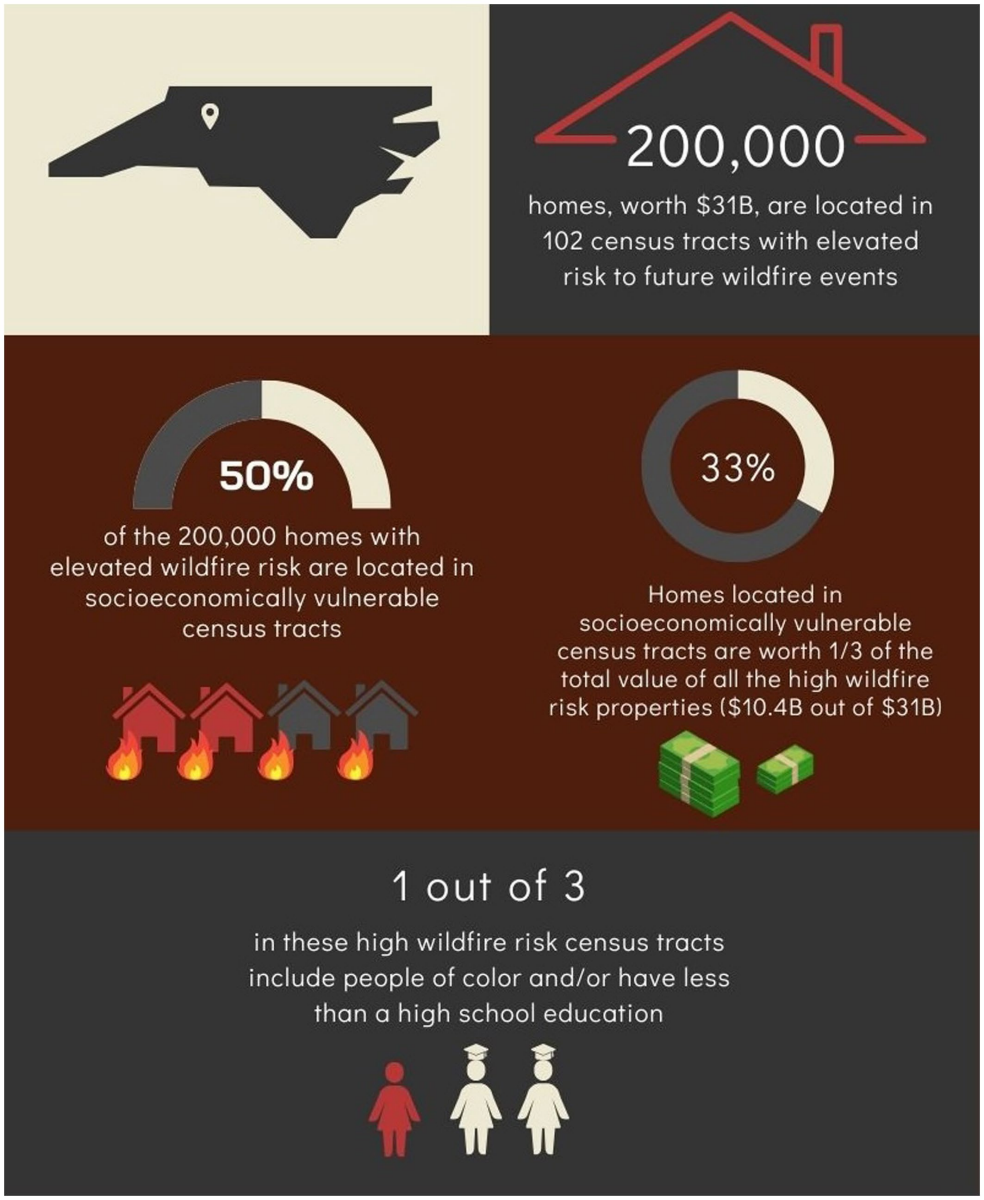
Summary of this study’s integrative WHP-SES analysis key findings. Three key findings, pertaining to projected monetary loss and housing damage as well as demographics, both economic and education-based, are shown in the figure.

A select view of these statistics is also provided in Figure 5 as an infographic.

### Dissemination of wildfire risk information via ENVIROSCAN

**Figure 6:**
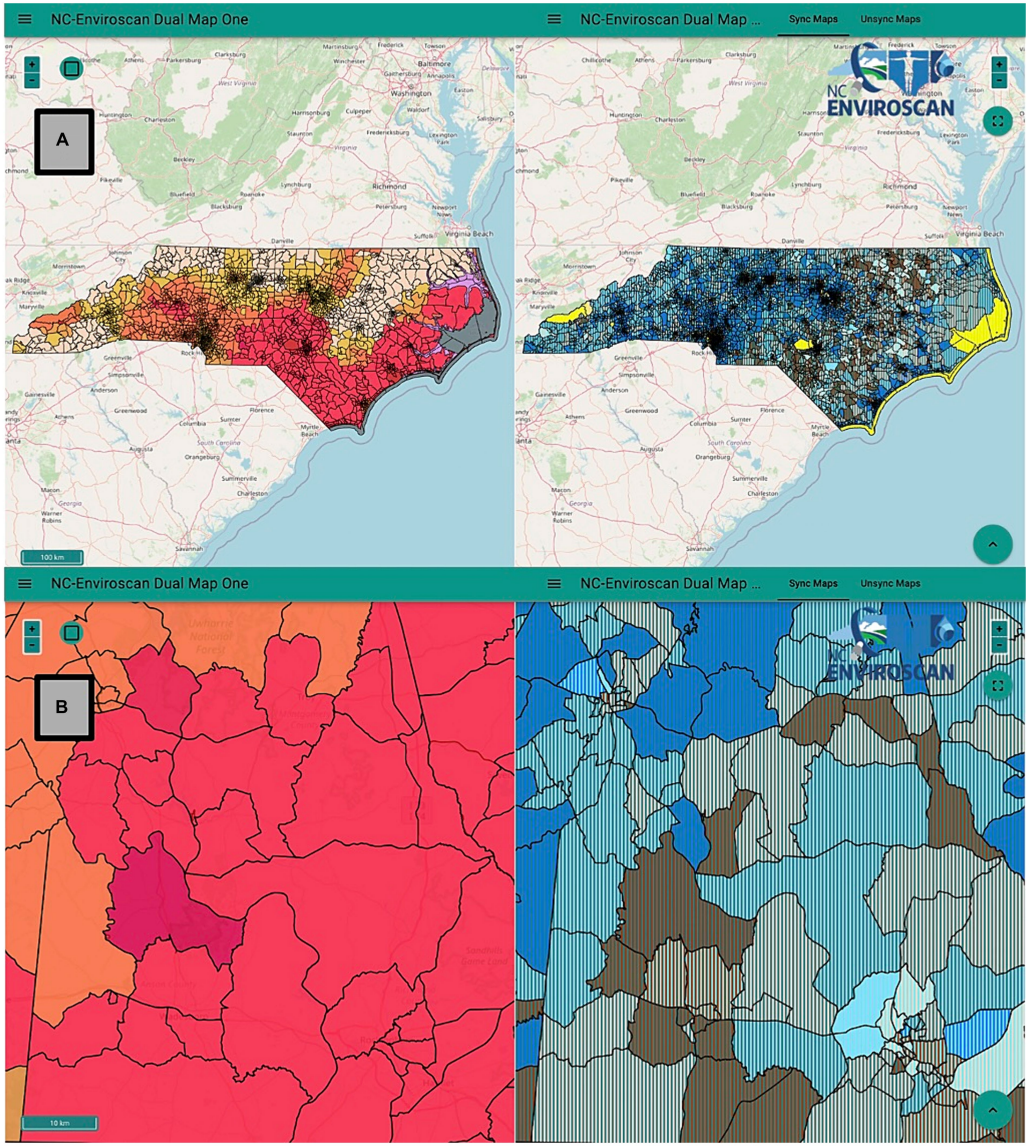
WHP and SES data dissemination through the publicly available application, ENVIROSCAN. **(A)** Screen view of the online application’s side-by-side feature. The left panel displays WHP values by census tract on a quintile scale, with values in the highest quintile category highlighted in dark red and those in the lowest quintile category highlighted in light yellow. The right panel combines both percent below the poverty level—the darkest blue represents the highest quartile category of poverty, and the lightest blue represents the lowest quartile category—and the Environmental Justice Index (EJI) National Scale Air Toxics Assessment Respiratory Hazard Index–the darker brown represents tracts in the highest quartile category, and the light brown represents tracts in the lowest quartile category (47). Tracts with blue and green coloration have the lowest values for both indicators, and tracts with brown and gold coloration have the highest values for both indicators. The online application allows users to display both features together on the same map, which allows for simple interpretation of unique datasets across the entire state. **(B)** Screen view of the application’s side-by-side feature zoomed in to focus on a block of census tracts located in Anson and Montgomery counties. The comparative map shows the WHP value of the census tracts—indicated by a dark red color—on the left side of the map, and both the level of poverty and the EJI Respiratory Hazard Index—represented by the brown and blue—in the interleaved map on the right.

One of this study’s goals was to disseminate findings of WHP distributions across NC via our public facing web portal, ENVIROSCAN (25). Our team integrated census tract-level WHP values into the application, which now displays WHP values across NC (Figure 6A). Select SES variables are also available within this application, allowing users to view these data side-by-side for easy comparison (Figure 6B). This application provides public health professionals and community members access to a wide range of environmental justice indicators such as heavy metals, SES variables, and health outcomes throughout the state.

## Discussion

This study examined trends in future wildfire risk throughout NC and identified areas of heightened public health concern due to underlying social vulnerability. Minoritized, low-income and/or socially excluded communities are already disproportionately experiencing the impacts of climate change (48); however, the extent to which wildfire risk in NC is unevenly distributed has yet to be examined. Filling this gap, this study presented several new findings. First, higher exposure risk (i.e., high WHP) was largely present in Southeastern NC. Second, integrative cluster analyses identified two clusters (Clusters 2 and 3) of socially vulnerable populations, primarily in Southeastern NC; one of these clusters (Cluster 3) had the highest WHP overall. Third, 102 census tracts with the highest WHP (>95th percentile) contain complex socioeconomic disparities and significant economic vulnerability to wildfire, as these regions were also located in the derived Clusters 2 and 3. Lastly, information on WHP and SES factors are disseminated to the public via the online mapping tool, ENVIROSCAN.

There is a notable concentrated region of high WHP values along the Southeastern part of the state, with high spatial autocorrelation to neighboring census tracts. Specifically, the three counties with the highest WHP were Richmond, Scotland, and Craven counties followed by Pender, Moore and Hoke counties. The high WHP in these counties is likely driven by the difference in land cover. Specifically, the vegetation in this eastern region is mainly shrubland/herbaceous and planted/cultivated, whereas the rest of the state is comprised of more forest and developed land (28). Additional factors that drive high WHP include amount of burnable land cover, frequency of historic fires, and resistance to wildfire control due to the common fuel types in an area (29). Identification of regions of increased exposure likelihood to wildfires is critical; and to further increase our understanding surrounding risk, it is important to consider both exposure to the hazard (i.e., WHP) with vulnerability to said hazard (49).

In this study we identified distinct regions throughout NC distinguished based upon SES attribute profiling. Two of the derived clusters, Clusters 2 and 3, were found to contain indicators of high SES vulnerability. Cluster 3 had the highest overall WHP, though both clusters include communities of greatest social vulnerability that will likely bear the brunt of economic effects in the face of wildfire events. Mirroring our findings, several other studies show that overall, there is not a large exposure disparity by race and/or ethnicity or SES factors with regards to wildfire risk (50). In fact, other studies using WHP across the country have found the highest hazards in largely White, high-income communities (20). Yet, socially vulnerable and excluded communities are more likely to suffer the greatest health and economic effects from wildfire for several reasons. First, preexisting conditions such as asthma and COPD that are exacerbated by wildfire exposure are associated with chronic poor air quality, which is recognized as being prevalent in poorer, BIPOC neighborhoods in NC (51). Second, indoor air quality issues are likely exacerbated in low SES communities during wildfire events. For example, socially and economically isolated/excluded groups are more likely to experience poor indoor air quality due to unsafe housing conditions, as well as crowding and financial barriers to household level protections such as air filters (52). These air quality issues likely become heightened as indoor air quality can be worse than outdoor air quality during wildfires (53). Furthermore, socially vulnerable communities have recently experienced an increased rate of smoke exposures due to wildfires just in the last decade, in comparison to other communities, nationwide (21).

Counties located within the socially vulnerable clusters, Cluster 2 and 3, included some of the poorest and historically marginalized communities largely located in Southeastern NC. Cluster 3 represented tracts with a high percentage of non-White residents and a high prevalence of poverty, as well as a substantial portion of the population without a high school education. Counties in this cluster included Anson, Columbus, Richmond, Robeson, and Scotland; of which, Richmond and Scotland also had the two highest WHP values within NC. Cluster 2 is comprised of counties such as Duplin and Sampson. Many of these counties are located in Southeastern NC which has a plagued history of environmental racism and is currently the site of numerous environmental injustices (54, 55). For example, confined animal feeding operations have demonstrated instances of polluting BIPOC and low-income communities (54), chemical manufacturers have leached PFAS into watersheds (56) and private wells have been contaminated with toxic metals (45) in these regions. Furthermore, Hoke, Robeson, and Scotland counties are homes of the Lumbee and Coharie tribe, and lands and waterways within these counties carry important cultural significance for the tribes (57). Thus, this research adds to the evidence base that the most socially vulnerable communities in NC (namely many counties in Southeastern NC) also face another increasing environmental threat: wildfires.

When we examined the 102 census tracts across NC with simply the highest WHP (>95th percentile), the socioeconomic characteristics of these communities painted a complex picture of populations at greatest exposure risk. Firstly, we found that there were over 200,000 homes, collectively worth $31billion, in these tracts, highlighting that the potential economic losses from wildfires in NC is substantial. However, we found that the relative vulnerability within these tracts was varied: 1 in 3 residents in these high-risk regions were people of color and/or those that have less than a high school education. Furthermore, around half of these tracts were within Cluster 2 or 3, the most socially vulnerable clusters. This underscores again that it is not solely exposure disparities (i.e., difference in WHP) but the combination of this hazard with vulnerability that will likely influence the effects of wildfires. Though NC is a state lower WHP values in comparison to Western states in the US, NC has a notably high proportion of communities with low socioeconomic status, creating a high level of vulnerability to the negative health effects of wildfire events (20).

Lastly, one of our goals was to ensure that data obtained during this study was communicated and made available to the public. ENVIROSCAN is an application that enables NC residents to visualize and compare environmental health and socioeconomic indicators (e.g., race, percent poverty, and percent multilingual). This web application is currently being expanded to states across the US; thus, we anticipate updated analyses through this tool to incorporate additional states outside of NC. The current project aimed to enhance the ENVIROSCAN database to allow users to examine how wildfire risk and sociodemographic factors are correlated. The ENVIROSCAN application was used to combine WHP and various sociodemographic indicators to elucidate geographical trends regarding wildfire risk, race/ethnicity, and socioeconomic status. Since the beginning of this project, ENVIROSCAN has been updated to include wildfire data, in the form of WHP values across NC. Users are now able to look at a map of NC that contains data on wildfire risk across the state, allowing NC residents to better prepare for future wildfire events. Our results also aid in informing resource management and community preparedness, while recognizing that further steps are necessary to translate these findings into on-ground actions by policymakers and community-level partners.

While among the first studies to identify social vulnerabilities to wildfire in NC, this study is not without limitations. The SES variables selected from the EJScreen provide only a partial picture of the social vulnerability. We recognize that many different tools and indices use different combinations of variables, such as the Social Vulnerability Index, Area Deprivation Index, Yost Index, Gini Coefficient, among others (58–61). Each of these indices inherently capture different aspects of vulnerability and may result in different findings (62). It is notable that the process of data selection itself is also a source of potential bias, and authors aim to update findings upon new data releases that more holistically capture socioeconomic vulnerability as the field continues to advance. Additionally, the usage of clustering, which is an unsupervised machine learning approach (40), was leveraged to determine co-occurrence patterns between SES variables and wildfire risk. It is notable that these methods do not capture causal relationships; rather, the selected methods represent unbiased pattern recognition approaches that are inherently exploratory, setting the stage for future research to further quantify likelihoods and future impacts of wildfire events alongside factors of climate change. Furthermore, the utilized SES variables represented estimates from 2015 to 2019, however these factors can vary over time and are possibly limited by the date of data generation and level of granularity.

Study findings open several avenues of further exploration. For example, future research could consider other indicators such as inflation, redlining, municipal underbounding, rental housing, and local public policy, which may also provide a more detailed explanation of disparities in wildfire exposure and economic/health vulnerability. Future research may also consider variations in wildfire risk over time, such as conducting temporal analyses, seeing as trends in wildfires have been evolving due to global climate change. Health outcome data such as hospital admissions, morbidity and mortality experienced after wildfire events, and prevalence of pre-existing conditions such as asthma would also further characterize and elucidate vulnerable populations within NC. Lastly, other indices exist to quantify aspects of future risks of wildfire events, such as the Wildland-Urban Interface (WUI). The WUI provides information relating a community or individual’s proximity to wildland and how that can influence one’s wildfire risk, and mainly focuses on population growth, the growing interface between urban areas and wildland, and subsequent wildfire risk (63). WHP was selected over other indices as it represents the most recently updated large-scale database relevant to geographic-based variables of wildfire event risk; however, WUI or other indices may impact additional insight into the growing risks of wildfire events.

## Conclusion

In conclusion, this study highlights regions across NC in which socially vulnerable clusters of populations are present, many of which coincide with areas of high WHP. It has been shown extensively in literature that being of a lower SES status puts one at a higher risk of certain health burdens, housing vulnerability, and economic stress. Residents of NC that fall into these categories (e.g., living below the poverty line) should have mechanisms in place to better protect themselves and be adequately prepared for likely impacts associated with climate change, including wildfire events. From the public health sector, initiative should be taken to provide education in these areas on the health risks associated with wildfire exposure and strategies implemented to mitigate and protect communities from wildfire events. Environmental justice is an ongoing issue, now being woven into climate justice as wildfires become more intense with the progression of climate change; more research must be done to determine which populations will need the most aid going forward in this changing environment. With these findings and plans in mind, government agencies and public health institutions can work to overcome the negative effects of climate injustice in NC and around the world.

## Data availability statement

The original contributions presented in the study are included in the article/Supplementary material (64). Further inquiries can be directed to the corresponding author.

## Author contributions

RW: Writing – original draft, Writing – review & editing, Data curation. AP: Writing – original draft, Writing – review & editing, Data curation, Formal analysis, Methodology, Visualization. EB: Writing – review & editing, Methodology. EM: Writing – review & editing. JF: Writing – review & editing, Investigation. CL: Writing – review & editing, Software. LE: Writing – review & editing, Conceptualization, Formal analysis, Methodology, Writing – original draft. RF: Writing – original draft, Funding acquisition, Resources. JR: Writing – original draft, Data curation, Funding acquisition, Methodology, Project administration, Resources, Supervision, Writing – review & editing.
